# Histopathologic characteristics and short-term outcomes of colorectal cancer in young Tunisian patients: one center's experience

**Published:** 2012-05-22

**Authors:** Mahdi Bouassida, Bilel Feidi, Bassem Mroua, Mohamed Fadhel Chtourou, Selim Sassi, Fathi Chebbi, Souheil Bouchtili, Mohamed Mongi Mighri, Hassen Touinsi, Mohamed Msaddak Azzouz, Sadok Sassi

**Affiliations:** 1 Mohamed Thahar Maamouri Hospital, Nabeul, Tunisia

**Keywords:** Colorectal cancer, young, Tunisia, surgery, survival

## Abstract

**Introduction:**

Colorectal carcinoma (CRC) is generally a disease of persons older than 40 years. Concerning younger patients, controversies still exist regarding features and prognosis of CRC. We performed this study to characterise CRC in young patients (≤40 years) as well as to evaluate short-term outcome in comparison with older patients (>40 years) with CRC.

**Methods:**

Clinical and histopathological parameters of 40 patients aged 40 years or less were compared with 240 patients aged more than 40 years.

**Results:**

In young patients, the minority suffered from hereditary cancer syndromes (0.4%). Furthermore, up to 87% of young patients denied any cancers in their families. Compared with older patients, young patients had more mucinous adenocarcinomas (32.5% vs. 11.5%; p=0.02), more venous invasion (p=0.021), more perineural invasion (p=0.028). For grading (p=0.42), lymphatic invasion (p=0.17) and tumor sites (p=0.46), no significant differences between young and older patients were found. Young patients had less post operative morbidity (p=0.039), less post operative mortality (0.029). Young and older patients had the same overall 1-year survival rates (p=0.24), and the same cancer-related 1-year survival rates (p=0.1).

**Conclusion:**

Tunisian patients present with colorectal cancer at a more advanced stage of the disease at younger ages compared to developed countries. The early detection of CRC followed by a sufficient oncologic treatment is crucial regardless of age. It is mandatory for all patients with suspicious symptoms to undergo early adequate diagnoses.

## Introduction

The incidence of colorectal cancer varies widely with higher incidence in North America, Australia, and northern and western Europe [1]. Developing countries have lower rates, particularly Africa and Asia. More than 90% of colorectal cancer cases occur in people aged 50 or older [2]. Nevertheless, colorectal cancer appears to be increasing among younger persons. The incidence of CRC at 40 years or younger varies between 1.6% and 23% [3, 4]. The highest incidences are in Asia and Middle East countries, and the lowest incidences are in Europe and in The United States. This incidence is still unknown in North Africa.

**Table 1:** Pathological characteristics of tumours

	≤40 years	>40 years	p
All patients	40	240	
**Tumour site**			
Colon	80%	82%	0.14
Rectum	20%	18%	
Histology of specimen			
Adenocarcinoma	77.5%	86.6%	
Mucinous adenocarcinoma	32.5%	11.6%	0.02
Others	0%	1.8%	
**Histological grade**			
Well-differenciated	42.5%	47.5%	
Moderatelly differenciated	25%	40.8%	
Indifferenciated	32.5%	11.6%	0.05
**Lymphatic invasion**			
N0	27.5%	36.66%	0.17
N1	72.5%	63.33%	
**Perineural invasion**			
Yes	47.5%	30.4%	0.028
no	52.5%	69.6%	
**Venous invasion**			
Yes	67.5%	48.75%	0.021
no	33.5%	51.25%	
**Liver metastasis**			
Yes	12.5%	14.5%	0.47
no	87.5%	85.5%	
**Peritoneal metastasis**			
Yes	7.5%	10.4%	0.41
no	92.5%	89.6%	
**Local R classification**			
R0	97.5%	85%	0.018
R1	2.5%	15%	
**Tumor stage**			
I	7.5%	13.33%	
II	17.5%	18.33%	
III	52.5%	49.5%	
IV	22.5%	18.84%	

**Table 2:** Treatment strategies

	≤40 years	>40 years
Palliative surgery	17.5%	22.9%
Curative surgery	82.5%	77.1%
**Emergent surgery**		
Yes	27.5%	24.16%
No	72.5%	76.84%
Right colectomy	30%	17.5%
Left colectomy	15%	14.6%
Subtotal colectomy	5%	6.25%
Total colectomy	5%	3.75%
Sigmoidectomy	7.5%	15.41%
Anterior resection	30%	26.66%
Abdomino-perineal resection	7.5%	5%
Stomie	0%	10.83%
Neoadjuvant treatment		
Radiotherapy	7.5%	3.75%
Chemoradiotherapy	2.5%	4.16%
**Adjuvant treatment**		
Chemotherapy	40%	27.5%
Radiotherapy	0%	0.45%
Chemoradiotherapy	5%	1.37%

Results of previous investigations, regarding young patients with CRC, reported more advanced stages of disease, and a worse survival in younger than in older patients. It is still unclear if these findings are associated with patient, tumor or treatment characteristics. Behind the remaining questions concerning this issue, we analysed the data of 280 patients treated at our institution within a 10-year period of time. We identified 14.2% of patients >40 years of age and compared their data with patients >40 years.

## Methods

### Patients

The records of 280 patients with colorectal cancer, who were referred between 2001 and 2010 to the Department Of Surgery, Hospital of Nabeul (north east of Tunisia), were retrospectively analysed. The age of 40 was used to separate the patients into two groups “Young” (aged under 40), and “old” (41 and above). Of these patients, 40 (14.2%) were younger than 40 years.

The clinical data of each of these patients were analysed for age at time of operation, sex, performance status, location of tumor, stage, tumor differenciation. Disease stage was determined according to the sixth edition of the TNM classification of the Union Internationale Contre le Cancer (UICC). Tumour classification of histomorphology follows the rules of the World Health Organization. All histopathological analyses were performed by a gastrointestinal histopathologist. Routine follow-up was carried out at 3-month intervals for 2 years and every 6 months thereafter for a total of 5 years. After completion of regular follow-up, patients or their family doctors were contacted by telephone or post at longer intervals.

### Tumour localization

The tumour localisation was classified in four groups: rectal cancers included tumours within a distance of 16 cm or less from the anal verge, measured with a rigid sigmoidoscope. All tumours above 16 cm from the anal verge were declared as colon cancers and subdivided in tumours of the sigmoid colon, left colon (including descending colon, left flexure and transverse colon) and right colon (including right flexure, ascending colon, cecum and appendix), according to the operation protocol. Colon resection was performed with a standardised regional lymph node dissection, and rectum resection with a total mesorectal excision, or partial mesorectal resection in upper third rectal carcinomas.

### Statistical analysis

All statistical analyses were evaluated using the statistical software: Statistical Package for Social Sciences (SPSS) for Windows (version 19.0, SPSS Inc., Chicago, IL, USA). Variables were compared by the chi-square test. A p value

## Results

### Incidence and patient's demography

Patients under the age of 40years, with colorectal cancer constituted 14.2% of 280 patients operated on for primary colorectal cancer. There were 20 (50%) female patients and 20 (50%) male patients in the group of young patients. This sex distribution was similar in the group of older patients, which included 101(42%) woman and 139 (58%) man. The median age of all patients with colorectal cancer was 59.2 years (range 14-96). In young patients, the median age was 32 years (range 14-40), and among the elderly was 63 years (range 41-96).

### Risk factors for developing CRC

In the young group, only one patient presented with risk factors for developing CRC (PAF), 4 patients had a first degree relative suffering from CCR. In 87.5% (n=35), the patient presenting at our institution was the first family member affected by any malignancy. In the older group, 2% (n=5) of patients presented with risk factors for developing CRC, like FAP (1.6%; n=4) and HNPCC (0.4%; n=1). 13 patients (5.4%) had a family history of CCR.

### Clinical presentation

The duration of symptoms at presentation in young patients (mean 1.8 months) was different from older patients (mean 4.2 months). All symptoms declared at admission to the hospital, except per-anal bleeding (22.5% in patients ≤40 years vs. 39.5% in patients >40 years; p=0.02), showed an equal distribution in patients under and over 40 years of age. The most frequent presenting complaints were a recent change in bowel habits (17.5% vs. 18.7%), abdominal pain (52% vs. 46%), weakness (27.5% vs. 34%), paradoxical diarrhea (7.5% vs. 10%) and constipation (17.5% vs. 23.75%). In most, symptoms were multiple, all these patients underwent colonoscopy and biopsies. On the other hand, there is no significant differences between young and older patients in emergency presentations, such as acute bowel obstruction (20% vs. 22%, p=0.47).

### Tumour localization

No significant differences between young and older patients were found. In young patients, 80% of the tumours were located in the colon and 20% in the rectum. In older patients, 82% of the tumours were located in the colon, and 18% in the rectum (p=0.46). Concerning colic tumours, the proportions of tumor localisation (right and left sided) were similar between the groups (p=0.14).

### Pathological characteristics of tumours

There were no significant differences between the groups concerning histopathology staging of tumours. In the younger age group, 25% were found to have stage I/II disease, 52.5% stage III and 22.5% with stage IV cancer. In the older age group, 31.75% were found to have stage I/II disease, 49.5% stage III and 18.75% with stage IV cancer. Young patients up to 40 years showed significant differences within the histological type (more mucinous adenocarcinomas, p=0.02), the differenciation degree (more undifferenciate tumors, p=0.05), the venous invasion (more venous invasion p=0.021), the perineural invasion (more perineural invasion, p=0.028). For lymphatic invasion (p=0.17), liver metastasis (p=0.47), and peritoneal metastasis (p=0.41), no significant differences between young and older patients were found. Young patients had more R0 resections (97.5% vs. 85%; p=0.018).

### Treatment strategies

27.5% of the young patients were operated in emergent conditions (24.1% of older patients were operated in emergent conditions, p=0.9). The operation was curative in 82.5% of cases for young patients vs. 77.5% of cases for older patients (p=0.83). In rectal carcinomas, the frequency of neoadjuvant treatment did not differ between younger and older patients (p=0.45). There was no significant difference in adjuvant or palliative postoperative treatment which was offered (p=0.087).

### Postoperative morbidity and mortality

Postoperative morbidity was significantly elevated in older patients (p=0.039). Postoperative complications (surgical and nonsurgical) occurred in 5 patients (12.5%) in the young, and in 70 patients (29.1%) in the elderly. In the older patients, the most common surgical complications were anastomotic leakage and fistulae (10.4% vs. 5% in younger patients). 15.8% of the older patients required a reoperation, and only 5% of the young patients were reoperated (p=0.045). Nonsurgical complications included mostly pulmonary embolism (3%) and cerebral stroke (1.66%). In the young patients, none of the complications was fatal, but in the older, 22 patients (9.1%) died postoperatively (p=0.029).

### Follow-up

Median follow-up in all patients was 14 (range: 1-200) months. Median follow-up in young patients and older patients were 24 and 13 months, respectively. Non specific late complications occurred in 10% of cases in both young and older patients. The most common complications, were acute bowel obstruction, and benign anastomotic stenosis. Overall 1-year survival rates in young and older patients were 88% and 94% (p=0.24). Cancer-related 1-year survival rates in young and older patients were 88% and 96% (p=0.1).

## Discussion

Over ten years, forty of 280 (14.28%) patients treated at the department of surgery of Nabeul (north east of Tunisia), comprised the young colorectal cancer group. Prevalence was comparable with most other reports from Asia: 10.1% in Taiwan [5], 18% in Istanbul [6], 19.7% in Sri Lankan [7] and 23% in Saudi Arabia [8]. Our figure was considerably more than that reported from the West: 2.8% in the United States [9], 3% in France [9] and 5.5% in New Zealand [10]. The high percentage reported in developing countries may be due, in part, to the higher population of younger people in these countries [11]. The median age of our patients was 59.2, it was higher than the rate described in other Arab countries such as Saudi Arabia [12]. However, the median age described here, was younger than the age described in most developed countries, and it was also younger than the age described in the Center of Tunisia [13]. Interestingly, the impact of risk factors for developing CRC was low in patients up to 40 years of age, with 2.5% patients suffering from FAP.

In the young, the familial background for any kind of cancer was analysed. Surprisingly, most patients (87.5%) had no known cancer either in their first or second-degree relatives. A recent pathologic study compared tissue samples from patients aged > and <40 years with colorectal cancer. It showed that there was an increase of a-methylacyl- CoA racemase expression noted, and several micro- RNAs were found to be more highly expressed.

The authors postulated that post-translational regulation of RNA may be important in the development of colorectal cancer, in younger patients [14, 15]. This emphasizes the need of increased awareness, about the incidence of CRC, even in persons younger than 40 years with a healthy familiar background, and probably the general screening starting at the age of 40 years needs to be re-discussed. The outcome of CRC treatment depends strongly on stage at diagnosis.

Early recognition of CRC in patients under age 40, without these risk factors, requires clinical awareness and aggressive pursuit of symptoms. We showed, in the present study, an equal distribution of symptoms, in patients under and over 40 years of age. The most frequent symptoms were a recent change in bowel habits (17.5%), abdominal pain (52%), weakness (27.5%), paradoxical diarrhea (7.5%) and constipation (17.5%). Only per-anal bleeding was more frequent in older population (p=0.02). Like the previous Tunisian's study [13], the location of the primary tumor was evenly distributed throughout the colon and rectum in the present sample. 80% of the tumors were located in the colon (vs.82% in the older patients; p=0.46). Nearly all articles addressing CRC in the young looking at histology of resected specimens, noted a higher prevalence of mucinous or poorly differentiated tumours including signet ring tumours [16–19]. In our study, we also found a significant higher amount of mucinous adenocarcinomas in young patients (p=0.02), more undifferenciate tumors (p=0.05), more venous invasion (p=0.021), and more perineural invasion (p=0.028), but grading of young patients did not vary significantly (p=0.45) compared with older patients. Certainly, postoperative survival was significantly better in younger compared with older patients, secondary to less comorbidity and less emergency operations. Less comorbidity is also responsible for the similar overall survival rates in the young patients. In fact, postoperative morbidity was significantly elevated in older patients (p=0.039), the rate of reoperations was also significantly elevated in this group (p=0.045), and consequently, we noted less post operative death in young patients (p=0.029). Overall 1-year survival rates in young and older patients were similar (p=0.24), even Cancer-related 1-year survival rates were similar (p=0.1).

We could not detect a previously described worse prognosis in young patients with CRC. Despite their unfavourable histopathology, young patients present with the same overall survival compared with older patients, as they are at a better health condition (compared in ASA), suffer from less postoperative complications and are more likely to tolerate toxicities associated with chemotherapy.

## Conclusion

Prevalence of CRC in young patients, in North East of Tunisia, is higher than the prevalence described in developed countries. Not only hereditary syndromes and specific risk factors are responsible for CRC in younger patients. There is a significant amount of young patients who present with the first and only malignancy in their families, and therefore inherited syndromes are unlikely in these cases. Despite their unfavourable histopathology, young patients present with the same overall survival compared with older ones. Outcome of younger patients could be improved if screening colonoscopy would start at 40 years, as young patients could be diagnosed at an earlier tumour stage. This is the first study, in North Africa, which tried to analyse characteristics of CRC in young patients. Our results must be confirmed by further multicentric studies.
